# Association between *FOXP3* polymorphisms and expression and neuromyelitis optica spectrum disorder risk in the Northern Chinese Han population

**DOI:** 10.1515/tnsci-2022-0337

**Published:** 2024-04-05

**Authors:** Jing Liu, Gaoning Wang, Jiahe Yang, Yulin Wang, Ruoyi Guo, Bin Li

**Affiliations:** Department of Neurology, The Second Hospital of Hebei Medical University, No. 215, Hepingxi Road, Shijiazhuang, 050000, China; The Key Laboratory of Neurology (Hebei Medical University), Ministry of Education, Shijiazhuang, Hebei 050000, China; Department of Neurology, The First Hospital of Qinhuangdao, Qinhuangdao, China

**Keywords:** Bruton tyrosine kinase, mRNA, neuromyelitis optica spectrum disorder, rs2232365, single nucleotide polymorphisms

## Abstract

**Background:**

Forkhead box P3 (*FOXP3*) plays a critical role in the pathogenesis of autoimmune disorders. In the present study, we genotyped three single-nucleotide polymorphisms, namely, rs2232365, rs3761548, and rs3761549, to determine the relationship between *FOXP3* polymorphisms and neuromyelitis optica spectrum disorder (NMOSD) susceptibility among the Northern Chinese Han population.

**Materials and methods:**

We genotyped single nucleotide polymorphisms at loci of the *FOXP3* gene (rs2232365, rs3761548, and rs3761549136) in 136 NMOSD patients and 224 healthy subjects using the multiplex SNaPshot technique. Allele, genotype, and haplotype frequencies were compared. qPCR was used to analyze the mRNA expression levels of *FOXP3* in the peripheral blood mononuclear cells of 63 NMOSD patients and 35 healthy subjects. Non-parametric tests were used to test the FOXP3 mRNA expression across the different groups.

**Results:**

The minor allele frequency (MAF) of G in rs2232365 was markedly lower in the NMOSD group than in the control group (odds ratio [OR] = 0.57, 95% confidence interval [95% CI]: 0.41–0.79, *p* = 0.001). Using genetic (codominant, dominant, and recessive) models and performing haplotype analyses, the MAF of G in rs2232365 was shown to be associated with protection against NMOSD in this population. Furthermore, haplotype analysis revealed that the haplotype GCT and the rs2232365, rs3761548, and rs3761549 alleles predicted protection against NMOSD (OR = 0.63, 95% CI = 0.41–0.97, *p* = 0.038). The proportions of the three genotypes of rs2232365 (*p* = 0.001) were not significantly different between the moderate-to-severe (Expanded Disability Status Scale (EDSS) ≥ 3 points) and mild (EDSS < 3 points) groups. Evidently, the proportion of patients with the AA genotype (64.3%) among the rs2232365 patients was significantly greater in the moderate-to-severe group than in the mild group (36.4%). However, the proportion of patients with the GG genotype (15.2%) among the rs2232365 patients was significantly greater in the mild group than in the moderate-to-severe group (2.9%). The mRNA expression of *FOXP3* was markedly greater in the NMOSD group than in the control group (*p* = 0.001). Nevertheless, acute non-treatment patients exhibited lower FOXP3 mRNA expression than healthy controls and patients in the remission group (*p* = 0.004 and 0.007, respectively).

**Conclusion:**

*FOXP3* polymorphisms and haplotypes are related to NMOSD susceptibility among the Han Chinese population. The minor allele G of *FOXP3* rs2232365 and the haplotype GCT are associated with protection against NMOSD. The GG genotype may decrease the severity of NMOSD, whereas the AA genotype is related to moderate-to-severe NMOSD. *FOXP3* mRNA expression is greater in patients with NMOSD than in healthy controls. However, it is decreased in acute non-treatment patients compared with healthy controls.

## Abbreviations

ANT: acute non-treatment group
AOGC: acute long-term oral glucocorticoid group
APQ4: anti-aquaporin-4
CD: Crohn’s disease
CNS: central nervous system
FOXP3: Forkhead box P3
IL: interleukin
MAF: minor allele frequency
mRNA: messenger RNA
MS: multiple sclerosis
NMOSD: neuromyelitis optica spectrum disorder
PBMCs: peripheral blood mononuclear cells
PCR: polymerase chain reaction
RA: rheumatoid arthritis
SLE: systemic lupus erythematosus
SNPs: single nucleotide polymorphisms
TGF-β: transforming growth factor-β
Tregs: regulatory T cells

## 1 Background

Neuromyelitis optica spectrum disorder (NMOSD) is a rare neurologic autoimmune disease and a type of central nervous system (CNS) autoimmune disorder that may lead to inflammatory demyelinating lesions in the spinal cord or optic nerves [1,2]. The incidence of NMOSD in China per 100,000 person-years was 0.278. In the northern Chinese cities of Heilongjiang and Shanxi Provinces, the crude incidence rates of NMOSD per 100,000 person-years are 0.155 and 0.425, respectively [46]. Humoral immunity plays a crucial role in the pathogenesis of NMOSD because anti-aquaporin-4 (APQ4) immunoglobulin G (IgG) autoantibodies can bind to astrocytes and promote complement- and antibody-dependent cell-mediated cytotoxic effects [3,4]. Furthermore, the upregulation of proinflammatory factors in the cerebrospinal cord and serum, as well as the infiltration of local and systemic AQP4-specific lymphocytes and T cells in the brain, can considerably facilitate cerebral injury during NMOSD [5–12]. However, the mechanism through which endogenous immunity limits inflammatory injury in NMOSD patients has largely not been identified. Moreover, additional studies are warranted to understand the specific pathogenic mechanism and etiology of NMOSD, although several potential environmental and genetic factors affecting NMOSD susceptibility were recently identified. Studies have reported that gene polymorphisms in interleukin (IL)-1 receptor-associated kinase 1 [13], B-lymphoid tyrosine kinase [14], signal transducer and activator of transcription 4 [15], proline-rich coiled-coil 2A [16], and cholesterol 7α-hydroxylase [17] are associated with NMOSD susceptibility. However, compared with those of other immune disorders, such as systemic lupus erythematosus (SLE), multiple sclerosis (MS), autoimmune thyroiditis, and rheumatoid arthritis (RA), genetic studies on NMOSD are relatively rare.

Forkhead box P3 (FOXP3) is a transcription factor and a member of the fork-winged helix family. It is encoded by *FOXP3* located on the X chromosome. *FOXP3* deletion combined with loss of regulatory T cells (Tregs) can accelerate inflammatory and autoimmune syndromes [18–20]. Furthermore, ectopic *FOXP3* expression results in the suppressive ability of CD4CD25^+^ T cells to prevent autoimmune gastritis and inflammatory bowel disease [19]. However, Tregs with *FOXP3* deficiency exhibit decreased expression of marker genes such as cytotoxic T lymphocyte protein 4, Epstein‒Barr virus-induced gene 3, interleukin (IL)-10, and ectonucleoside triphosphate diphosphohydrolase 1; in contrast, T effector cytokine genes such as cytokine interferon gamma, tumor necrosis factor alpha, IL-4, and IL-17 are expressed [21–24]. In Scurfy mice, the FOXP3 protein, which lacks the forkhead domain, is expressed owing to a frameshift mutation in *Foxp3* [25]. Several additional loss-of-function *Foxp3* mutations have been discovered in patients who develop immune dysregulation, polyendocrinopathy, enteropathy, or X-linked inheritance syndrome [26,27]. Genetic mutations in *Foxp3* generally occur along with functional Treg loss, resulting in different autoimmune disorders. Previous studies have suggested that NMOSD susceptibility is associated with defects in naive Tregs [28,29]. Moreover, the promoter region of the FOXP3 gene contains three well-documented single nucleotide polymorphisms (SNPs) (SNPrs2232365, rs3761548, and rs3761549), which have been confirmed to be associated with the development of various autoimmune diseases, including MS, Graves’ disease, cancer, vitiligo, and ulcerative colitis [44,45,47–49]. Notably, these FOXP3 promoter SNPs can directly lead to changes in the binding of transcription factors, affecting the transcriptional activity of FOXP3 and its protein expression [50–53], which in turn may have an impact on the onset of autoimmune diseases.

As a result, in the present study, we determined whether *FOXP3* SNPs at the above loci (rs2232365, rs3761548, and rs3761549) can predict NMOSD susceptibility in the Han Chinese population. The relationships between FOXP3 alleles, genetic models, linkage disequilibrium (LD), and haplotypes and NMOSD were determined. Simultaneously, genotypes were stratified based on AQP4–IgG status and clinical manifestations. Finally, FOXP3 messenger RNA (mRNA) expression was detected in peripheral blood mononuclear cells (PBMCs) collected from patients with NMOSD and healthy Han Chinese people. To the best of our knowledge, our study is the first to explore the polymorphism of FOXP3 and its susceptibility to NMOSD in the northern Han Chinese population. The findings of this study increase the known spectrum of FOXP3-associated diseases and may provide some reference value for future gene modification treatments for NMOSD.

## 2 Materials and methods

### 2.1 Study participants

A total of 136 patients with NMOSD (11 men and 125 women) were recruited from the Department of Neurology, Second Hospital of Hebei Medical University, between December 2020 and March 2023. Furthermore, 224 normal individuals (207 women and 17 men) were recruited from a physical examination center. The participants were northern Han Chinese. NMOSD is a rare neurologic autoimmune disease. The incidence of NMOSD in China per 100,000 person-years was 0.278 [46]. Therefore, our study sample size is relatively limited. Patients with NMOSD who fulfilled the 2015 diagnostic criteria for NMOSD were recruited [30]. The exclusion criteria were as follows: had (a) other underlying autoimmune disorders, such as RA, SLE, or thyroid disorders, or (b) chronic disorders, such as tumors, hypertension, cerebrovascular disorders, or diabetes. Informed consent was obtained from all participants before starting the study. Patient information, including age, sex, onset age, AQP4 status, initial symptoms, clinical presentations, disease history, and medication history, was collected using a standard case report form.

### 2.2 Characteristics of the participants

Table S2 presents the characteristics of the participants. In total, 360 participants were enrolled for SNP analysis: 224 normal subjects (92.4% women) and 136 participants with NMOSD (91.9% women). The average ages of the participants in the NMOSD and control groups were 45.53 ± 14.15 and 43.77 ± 11.60 years, respectively; these findings suggest that there were no significant differences in age or sex between the two groups (*p* > 0.05). The average age of onset of NMOSD was 40.58 ± 12.96 years. In addition, 107 patients (78.68%) tested positive for serum anti-AQP4 antibodies. Optic neuritis was observed in 52 (38.24%) patients, acute myelitis in 57 (41.91%) patients, and brain and mixed attacks in 27 (19.85%) patients (Table S2 in the supplementary materials).

### 2.3 DNA extraction and genotyping

Briefly, 3–4 mL of peripheral venous blood was collected from patients with NMOSD and controls into ethylenediaminetetraacetic acid anticoagulation tubes. Thereafter, genomic DNA was extracted using a Blood Genomic DNA Extraction Kit (Gu De, Guangzhou, China) according to the specific instructions. DNA samples were stored at −80°C until subsequent analysis. Three FOXP3 genetic variants were chosen based on previous studies on additional autoimmune diseases (rs2232365, rs3761548, and rs3761549) and were identified via the PubMed database. Gene genotyping was performed using the SNaPshot SNP method, primarily involving polymerase chain reaction (PCR) amplification, followed by purification of PCR products. This mixture was subsequently subjected to an extension reaction, after which 1 µL of the extension product was taken, mixed with 9 µL of HiDi formamide, denatured at 95°C for 3 min, immediately placed in an ice bath, and finally loaded onto a sequencer for processing. Genotyping was performed using a Snapshot Kit (ABI, USA). Table S1 lists the sequences of primers used. Table S1 shows the detailed PCR conditions and representative peak charts used in the supplementary materials.

### 2.4 qPCR for *FOXP3* mRNA

Fasting venous blood (5 mL) was obtained from each participant to isolate PBMCs. Total RNA was extracted using total RNApure reagent (Servicebio, Wuhan, China) according to the specific instructions. The Prime Script™ RT reagent Kit was used to prepare cDNA from the RNA samples by using gDNA Eraser (Servicebio) according to specific protocols. qPCR mix (Servicebio) was used, and qPCR was performed using an ABI 7300 system. The reaction system comprises 2× Universal Blue SYBR Green qPCR Master Mix (10.0 μL), forward primer (10 μm) (0.4 μL), reverse primer (10 μm) (0.4 μL), template (2 μL), followed by addition of nuclease-free water to make a total volume of 20 μL. The PCR amplification conditions were 10 min at 95°C, 15 s at 95°C, and 1 min at 60°C for 40 cycles; followed by a final extension for 10 min at 72°C. Upon obtaining the cycle threshold (CT) value, calculate 2^–△△CT^, representing the relative quantification value of the target gene expression. Standardize and proceed with non-parametric tests for statistical analysis. Primer information is provided in the supplementary materials.

### 2.5 Statistical analysis

Demographic, clinical, and laboratory data are presented as mean ± standard deviation; on the other hand, frequencies are presented as numbers and percentages. Differences in the average values were compared using the unpaired Student’s *t*-test for both groups, whereas the chi-square test and Fisher’s exact test were used to analyze categorical data. Furthermore, the chi-squared test was used to calculate the Hardy–Weinberg equilibrium (HWE). The SNPs that significantly deviated from HWE (*p* < 0.05) were excluded. Using diverse genetic models (codominant, dominant, and recessive), logistic regression analysis was conducted to analyze the relationships after adjusting for sex and age. Furthermore, SHEsis (http://analysis2.bio-x.cn/myAnalysis.php), an online software package, was used for the HWE test, LD, and haplotype analysis. The SNPStats tool (https://www.snpstats.net/start.htm) was used to construct the haplotypes while analyzing the relationships among the associated factors. *FOXP3* mRNA expression was compared between the two groups using the non-parametric Mann–Whitney *U* test (non-normal distribution). In contrast, *FOXP3* mRNA expression was compared across the three groups using the Kruskal–Wallis (non-normal) test and Dunn’s test. The statistical analysis was performed using SPSS 25.0 (IBM Corp., Armonk, NY, USA). A two‐tailed *p* < 0·05 was used to indicate statistical significance.


**Ethical approval:** The research related to human use has been complied with all the relevant national regulations, institutional policies and in accordance the tenets of the Helsinki Declaration, and has been approved by the authors’ institutional review board or equivalent committee. This study was approved by the Ethics Committee of the Second Hospital of Hebei Medical University (No. 2021-R513).
**Informed consent:** Informed consent has been obtained from all individuals included in this study.

## 3 Results

### 3.1 Genotype and allele frequency distributions of the FOXP3 polymorphisms in the NMOSD and normal groups

The distribution of FOXP3 allele frequencies conformed to HWE in the NMOSD and control groups (*p* > 0.05). Furthermore, the minor allele frequency (MAF) of the three *FOXP3* SNPs was >5%. Table 1 presents the genotyping quality results.

**Table 1:** Data from the quality evaluation for genotyping

Test of HWE MAF (present‐study data)				
SNP groups		*p*‐value	Allele	Frequencies
rs2232365	NMOSD	0.826238	G	0.290
	Control	0.171883	G	
rs3761548	NMOSD	0.840396	A	0.217
	Control	0.197898	A	
rs3761549	NMOSD	0.248432	T	0.154
	Control	0.201864	T	

Abbreviations: Hardy‒Weinberg equilibrium (HWE); MAF: minor allele frequency.

Table 2 displays the FOXP3 allele and genotype distributions of rs2232365, rs3761548, and rs3761549 in both groups. The NMOSD group was not significantly associated with rs3761548 or rs3761549 (*p* > 0.05). Nevertheless, the frequency of the rs2232365 genotype was significantly different between the NMOSD group and the control group. In addition, the MAF G in rs2232365 was lower in the NMOSD group (0.29) than in the control group (0.42); this difference was strongly associated with a lower NMOSD risk (odds ratio [OR] = 0.57, 95% confidence interval [CI]: 0.41–0.79, *p* = 0.001).

**Table 2:** Genotypes and allele frequencies of the Foxp3 polymorphisms in the NMOSD patients and controls

			HCs	NMOSD	OR	
Gene SNP	Model	Genotype	*n* (%)	*n* (%)	95% CI	*p*‐values
rs2232365(A/G)	Alleles	A	261 (0.58)	193 (0.71)	1	**0.001**
		G	187 (0.42)	79 (0.29)	0.57 (0.41–0.79)	
	Codominant	A/A	81 (0.36)	69 (0.51)	1.00	**0.004**
		A/G	99 (0.44)	55 (0.40)	0.65 (0.41–1.03)	
		G/G	44 (0.20)	12 (0.09)	0.32 (0.16–0.66)	
	Dominant	A/A	81 (0.36)	69 (0.51)	1	**0.007**
		A/G-G/G	143 (0.64)	67 (0.49)	0.55 (0.35–0.85)	
	Recessive	A/A-A/G	180 (0.80)	124(0.91)	1	**0.005**
		G/G	44 (0.20)	12 (0.09)	0.40 (0.20–0.79)	
	Overdominant	A/A-G/G	125 (0.56)	81 (0.60)	1	0.470
		A/G	99 (0.44)	55 (0.40)	0.85 (0.55–1.32)	
rs3761548(A/C)	Alleles	A	102(0.23)	59 (0.22)	1	0.737
		C	346 (0.77)	213 (0.78)	0.94 (0.65–1.35)	
	Codominant	C/C	137 (0.61)	83 (0.61)	1	0.680
		A/C	72 (0.32)	47 (0.35)	1.08 (0.68–1.71)	
		A/A	15 (0.07)	6 (0.04)	0.69 (0.26–1.87)	
	Dominant	C/C	137 (0.61)	83 (0.61)	1	0.940
		A/C-A/A	87 (0.39)	53 (0.39)	1.02 (0.65–1.58)	
	Recessive	C/C-A/C	209 (0.93)	130 (0.96)	1	0.420
		A/A	15 (0.07)	6 (0.04)	0.67 (0.25–1.79)	
	Overdominant	C/C-A/A	152 (0.68)	89 (0.65)	1	0.640
		A/C	72 (0.32)	47 (0.35)	1.11 (0.71–1.76)	
rs3761549(C/T)	Alleles	C	363(0.81)	230(0.85)	1	0.228
		T	85(0.19)	42(0.15)	1.28 (0.86–1.92)	
	Codominant	C/C	150 (0.67)	99 (0.73)	1	0.500
		C/T	63 (0.28)	32 (0.24)	0.77 (0.47–1.26)	
		T/T	11 (0.05)	5 (0.04)	0.70 (0.23–2.08)	
	Dominant	C/C	150 (0.67)	99 (0.73)	1	0.240
		C/T-T/T	74 (0.33)	37 (0.27)	0.76 (0.47–1.21)	
	Recessive	C/C-C/T	213 (0.95)	131 (0.96)	1	0.600
		T/T	11 (0.05)	5 (0.04)	0.75 (0.25–2.21)	
	Overdominant	C/C-T/T	161 (0.72)	104 (0.77)	1	0.330
		C/T	63 (0.28)	32 (0.24)	0.78 (0.48–1.28)	

The genotypic frequency data were adjusted for age and sex using logistic regression analyses.

The results of genotypic frequencies using logistic regression analyses.

Abbreviations: 95% CI, 95% confidence interval; HCs, healthy controls; NMOSD, neuromyelitis optica spectrum disorders; OR, odds ratio.

The values in bold are considered to be significant.

The SNP distribution was examined using four genetic models (codominant, dominant, recessive, and overdominant). The GG genotype of rs2232365 markedly decreased NMOSD susceptibility in the codominant (G/G vs A/A, OR = 0.32, 95% CI = 0.16–0.66, *p* = 0.004), dominant (A/G + G/G vs A/A, OR = 0.55, 95% CI = 0.35–0.85, *p* = 0.007), and recessive (G/G vs A/G + A/A, OR = 0.40, 95% CI = 0.20–0.79, *p* = 0.005) models. These findings suggest the protective role of the GG phenotype against NMOSD. Additional details are presented in Table 2.

### 3.2 LD and haplotype analyses

**Figure 1:**
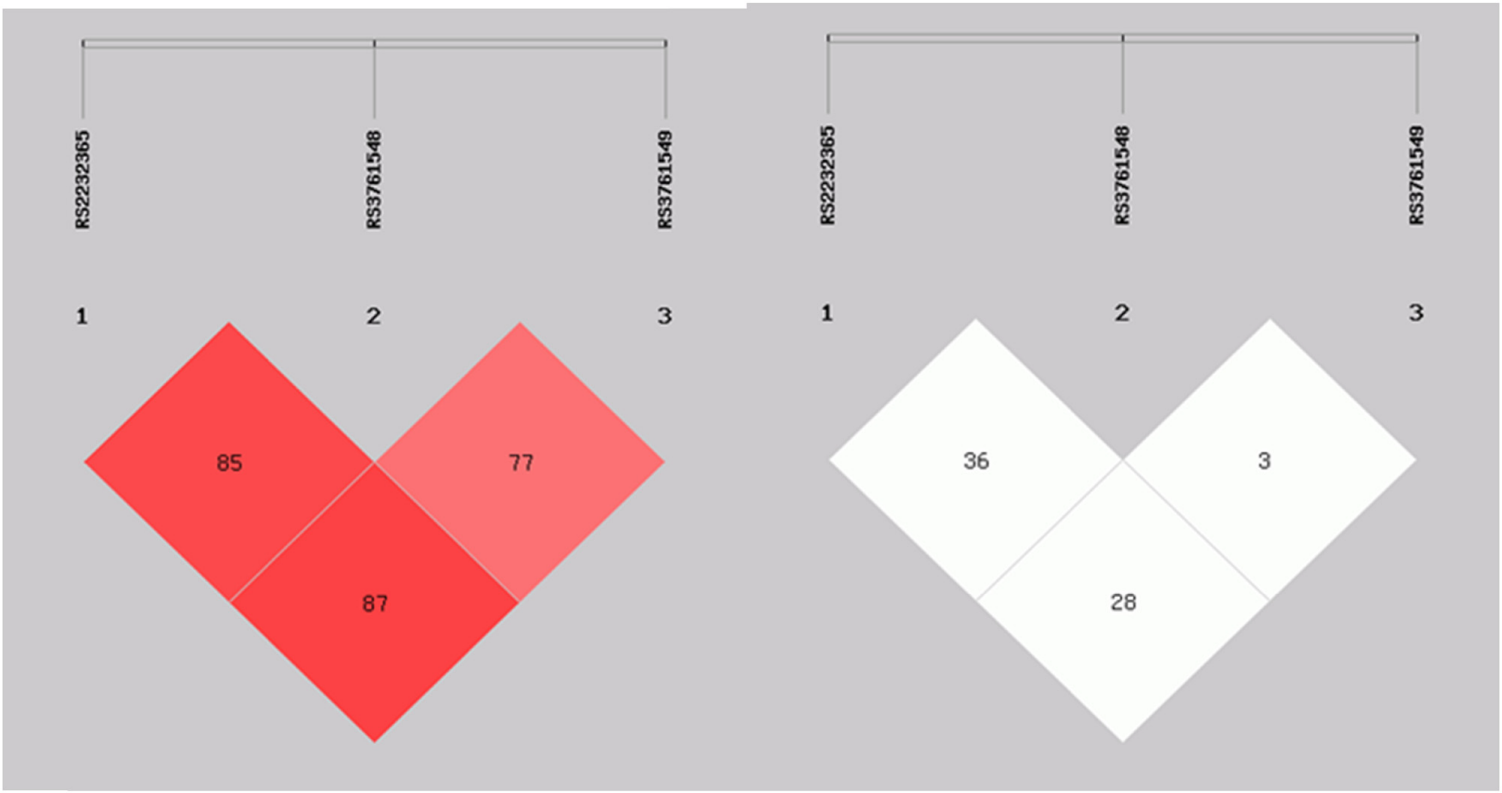
Logistic chosen for haplotype analysis: rs2232365, rs3761548, and rs3761549. LD patterns in FOXP3. *D*′ (a) and *r* ^2^ (b) mean LD coefficients of the three SNPs. Each block represents the LD relationship between two SNPs. The rs2232365 and rs3761548 variants exhibited LD for *D*′ = 0.859 and *r* ^2^ = 0.363. The rs2232365 and rs3761549 variants exhibited LD for *D*′ = 0.878 and *r* ^2^ = 0.282. The rs3761548 and rs3761549 variants exhibited LD for *D*′ = 0.774.

Among the three SNPs investigated in this study, we determined LD based on *D*′ and *r* ^2^ values. rs2232365 and rs3761548 exhibited LD (*D*′ = 0.859, *r* ^2^ = 0.363) (Figure 1). Furthermore, rs2232365 exhibited LD with rs3761549 (*D*′ = 0.878, *r* ^2^ = 0.282). In addition, rs3761548 and rs3761549 exhibited LD (*D*′ = 0.774). Next, we elucidated FOXP3 haplotypes based on the LD among the three SNPs. The following haplotypes were constructed in the order of rs2232365, rs3761548, and rs3761549; haplotypes (ACT, AAT, GCC, GAT) with a frequency of <3.0% in both groups were excluded. Table 3 presents the remaining three haplotypes. The GCT haplotype was significantly different between the NMOSD group and the control group. In particular, the GCT haplotype with the minor G allele of rs2232365 may be associated with protection against NMOSD (OR = 0.63, 95% CI = 0.41–0.97, *p* = 0.038) and may have protected against NMOSD susceptibility.

**Table 3:** Haplotype analysis of FOXP3 polymorphisms in NMOSD patients and controls

Haplotype	Case (%)	HCs (%)	*p*‐value	OR (95% CI)
ACC	0.6432	0.5804		1
GAC	0.1641	0.2277	0.074	0.69 (0.46–1.04)
GCT	0.1264	0.1875	0.038	0.63 (0.41–0.97)
Rare	*	*	0.110	4.00 (0.75–21.36)

Abbreviations: HCs, healthy controls; 95% CI, 95% confidence interval; OR, odds ratios; * represents rare haplotype.

### 3.3 Stratification analyses of FOXP3 polymorphisms based on the clinical characteristics of patients with NMOSD

Statistical analysis was performed to assess the role of FOXP3 SNPs in the analyzed clinical features (AQP4 status and onset of symptoms). Allele and genotype frequencies did not significantly differ after stratifying the clinical features (Table 4). Patients with NMOSD were divided into moderate-to-severe (Expanded Disability Status Scale [EDSS] ≥3 points) and mild (EDSS <3 points) groups [31], with average ages of 48.31 ± 14.42 and 44.59 ± 15.19 years, respectively. The two groups did not exhibit any significant difference in average age (*p* = 0.145), and the percentages of women in the two groups were 92.9 and 90.0%, respectively, with no significant difference in sex (*p* = 0.677). However, in the rs2232365 subgroup, the percentages were significantly different between the two groups (*p* = 0.001), with genotype AA accounting for 64.3% of the participants in the moderate-to-severe subgroup, which was markedly greater than that in the mild subgroup (36.4%). In contrast, in the mild group, the GG genotype accounted for a noticeably greater percentage (15.2%) of the population in the mild group than in the moderate-to-severe group (2.9%).

**Table 4:** Association of FOXP3 rs2232365 rs3761548 and rs3761549 with clinical characteristics of patients with NMOSD

	rs2232365				rs3761548				rs3761549			
Clinical characteristics	AA	AG	GG	*p*	CC	AC	AA	*p*	CC	CT	TT	*p*
AQP4‐IgG positive *n* (%)	56 (52.3)	44 (41.1)	7 (6.5)	0.24	69 (64.5)	34 (31.8)	4 (3.7)	0.28	77 (72)	26 (24.3)	4 (3.7)	0.91
AQP4‐IgG negative *n* (%)	13 (44.8)	11 (37.9)	5 (17.2)		14 (48.3)	13 (44.8)	2 (6.9)		22 (75.9)	6 (20.7)	1 (3.5)	
OR (95%CI)	1	1.08 (0.44–2.64)	3.08 (0.84–11.25)		1	1.88 (0.80–4.45)	2.46 (0.41–14.79)		1	0.81 (0.30–2.21)	0.87 (0.09–8.24)	
Onset syndromes, *n* (%)												
Optic neuritis	29 (42.0)	23 (41.8)	3 (25.0)	0.903	30 (36.1)	21 (44.7)	1 (16.7)	0.553	40 (40.4)	11 (34.4)	1 (20.0)	0.721
Acute myelitis	26 (37.7)	21 (38.2)	7 (58.3)		37 (44.6)	17 (36.2)	3 (50.0)		39 (39.4)	14 (43.8)	4 (80.0)	
Brain attacks	8 (11.6)	7 (12.7)	1 (8.3)		9 (10.8)	5 (10.6)	2 (33.3)		12 (12.1)	4 (12.5)	0 (0.0)	
Mix attacks	6 (8.7)	4 (7.3)	1 (8.3)		7 (8.4)	4 (8.5)	0 (0.0)		8 (8.1)	3 (9.4)	0 (0.0)	
EDSS score EDSS ≥3 EDSS ＜3	45 (64.3) 24	23 (32.9) 32	2 (2.9) 10	0.001	49 (70.0) 34	19 (27.1) 28	2 (2.9) 4	0.083	54 (77.1) 45	15 (21.4) 17	1 (1.4) 4	0.269
	(36.4)	(48.5)	(15.2)		(51.5)	(42.4)	(6.1)		(68.2)	(25.8)	(6.1)	

Abbreviations: 95% CI, 95% confidence interval; OR, odds ratios; NMOSD, neuromyelitis optica spectrum disorders; APQ4: anti-aquaporin-4; EDSS, Expanded Disability Status Scale.

### 3.4 FOXP3 mRNA expression in the NMOSD and control groups

**Figure 2:**
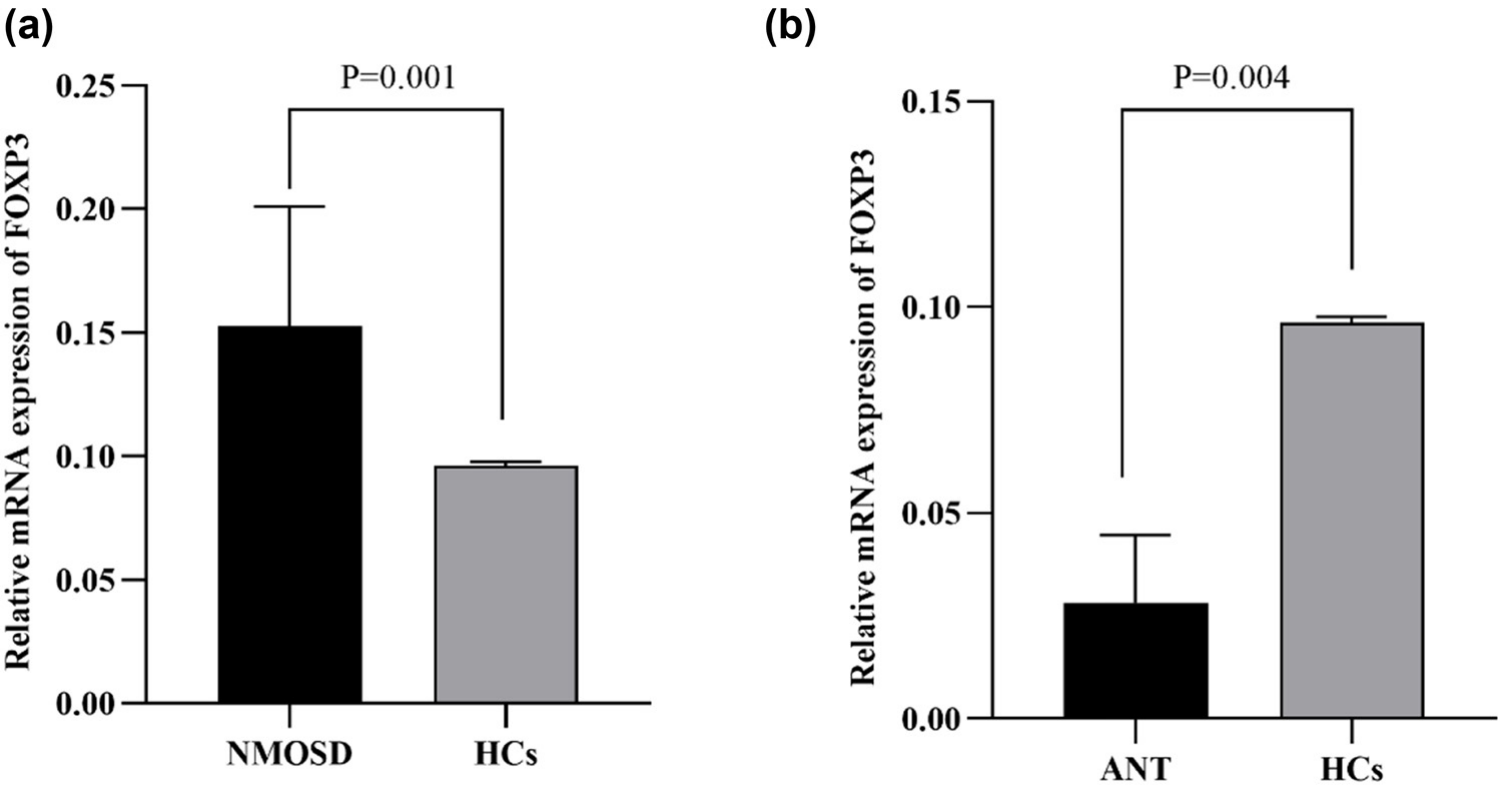
Relative mRNA expression of FOXP3 in the NMOSD, HCS, and ANT groups. FOXP3 mRNA levels in PBMCs. The *p*‐value was calculated by comparisons of individuals’ relative expression using the 2^−ΔΔCt^ method. RT‐PCR was used to determine the relative quantification of the FOXP3 mRNA expression level in the PBMCs of HCs and NMOSD patients. (a) FOXP3 mRNA expression was increased in the total NMOSD patients (*n* = 63) compared with that in the healthy controls (*n* = 35) (*p* = 0.001). (b) FOXP3 mRNA expression was lower in the acute untreated group (*n* = 6) than in the healthy control group (*n* = 35) (*p* = 0.004). *p* < 0.05 according to the non-parametric Mann–Whitney *U* test (non‐normality). HCs, healthy controls; ANT, acute non‐treatment. Each *p*‐value was corrected for age and sex.

A total of 63 NMOSD samples were collected to measure *FOXP3* mRNA expression in PBMCs (3 men and 60 women; average age of 45.56 ± 13.34 years). Furthermore, 35 healthy control samples were randomly collected (3 men and 32 women; average age of 46.86 ± 14.33 years). No obvious differences in age or sex were noted between the two groups (*p* = 0.653 and 0.663, respectively). Compared with those in the control group, *FOXP3* mRNA expression in the NMOSD group was markedly greater (*p* = 0.001) (Figure 2a). Moreover, the 63 NMOSD patients were classified into three groups: (1) the acute non-treatment group (ANT), in which six acute-phase patients were admitted with no previous medication before blood sampling or drug administration for 1 month before disease onset; (2) the acute long-term oral glucocorticoid group (AOGC), in which 20 acute-phase patients received routine long-term oral glucocorticoids before acute onset; and (3) the remission group, in which 37 stable-phase patients received long-term oral drugs (such as glucocorticoids, mycophenolate mofetil, and azathioprine). When comparing ANT patients with age- and sex-matched healthy controls, the ANT group had decreased *FOXP3* mRNA expression (*p* = 0.004) (Figure 2b).

**Figure 3:**
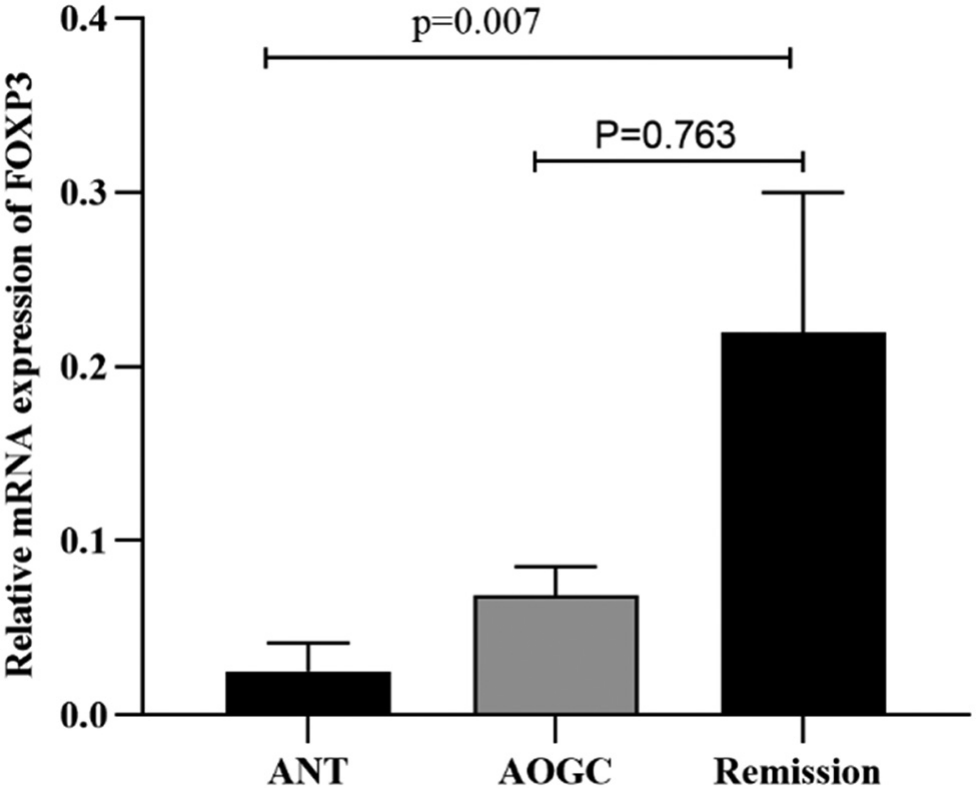
Relative mRNA expression of FOXP3 in the ANT, AOGC, and remission groups. The 63 NMOSD patients were divided into three groups: ANT (*n* = 6), AOGC (*n* = 20), and remission (*n* = 37). The expression levels of FOXP3 were arranged in ascending order among the three groups: ANT<AOGC<remission group. FOXP3 mRNA expression in the ANT was lower than that in the other two groups (*p* = 0.009). The proportion of patients in the ANT group was lower than that in the remission group (*p* = 0.007). There was no significant difference in the AOGC score compared to that in the remission group (*p* = 0.763). We used the Kruskal–Wallis test with Dunn’s correction (non‐normality) for three-group comparisons. All *p* values were corrected for age and sex.

**Figure 4:**
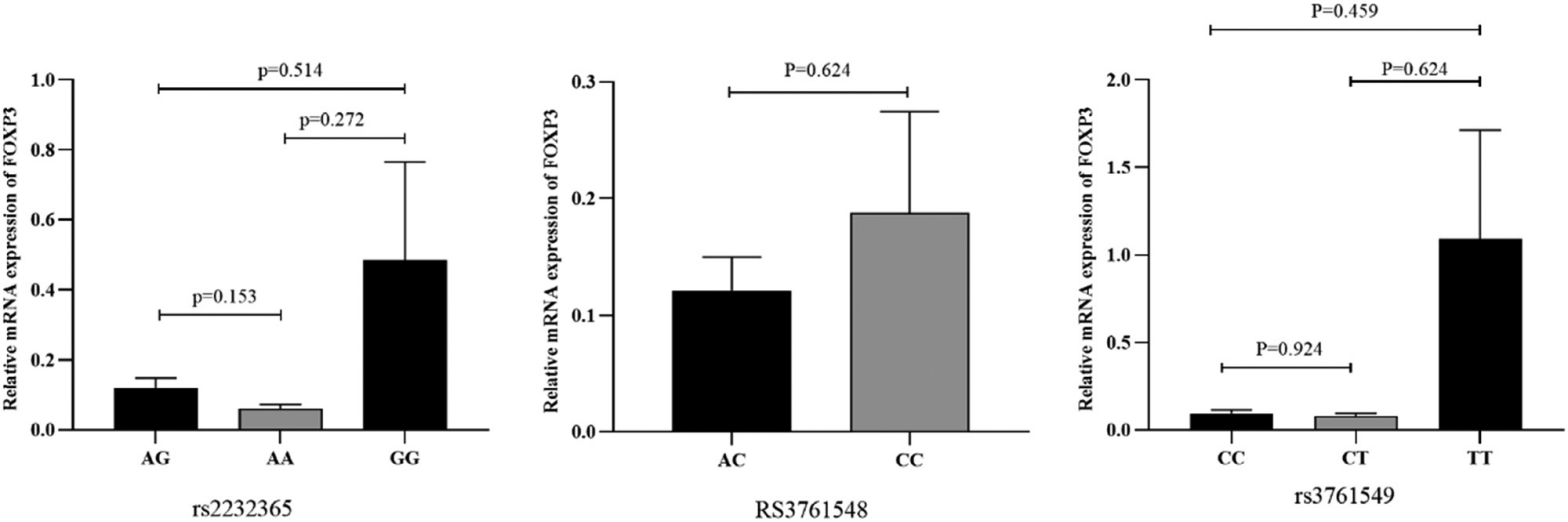
FOXP3 mRNA expression levels in patients with different genotypes of rs2232365, rs3761548, and rs3761549 among the NMOSD patients. The data of 61 NMOSD patients, including rs2232365 (AA 25, AG 27, and GG 9), rs3761548 (AC 26, CC 34, and AA1), and rs3761549 (CC 44, CT 13, and TT 4), were analyzed. The Mann–Whitney *U* test (non‐normality) was applied for the statistical analysis. There was no observable significant variation in FOXP3 mRNA expression in NMOSD patients with different genotypes (*p* > 0.05). Each *p*-value was corrected for age and sex.

When the three groups were adjusted for age and sex, *FOXP3* expression followed the order of ANT < AOGC < remission group, from the lowest to the highest. Moreover, *FOXP3* mRNA expression was lower in the ANT group than in the other two groups (*p* = 0.009). In addition, the ANT group exhibited significant differences compared with the remission group (*p* = 0.007). However, compared with those in the remission group, the AOGC group did not exhibit any significant differences; nevertheless, *FOXP3* mRNA expression was lower in the AOGC group (*p* = 0.763) (Figure 3). In addition, *FOXP3* mRNA expression was not significantly different among the diverse genotypes of NMOSD (*n* = 61). Nevertheless, patients with NMOSD who carry the AA genotype may exhibit decreased *FOXP3* expression compared with those who carry the AG and GG genotypes (*p* > 0.05) (Figure 4).

### 3.5 Gender analysis of FOXP3 polymorphisms

**Figure 5:**
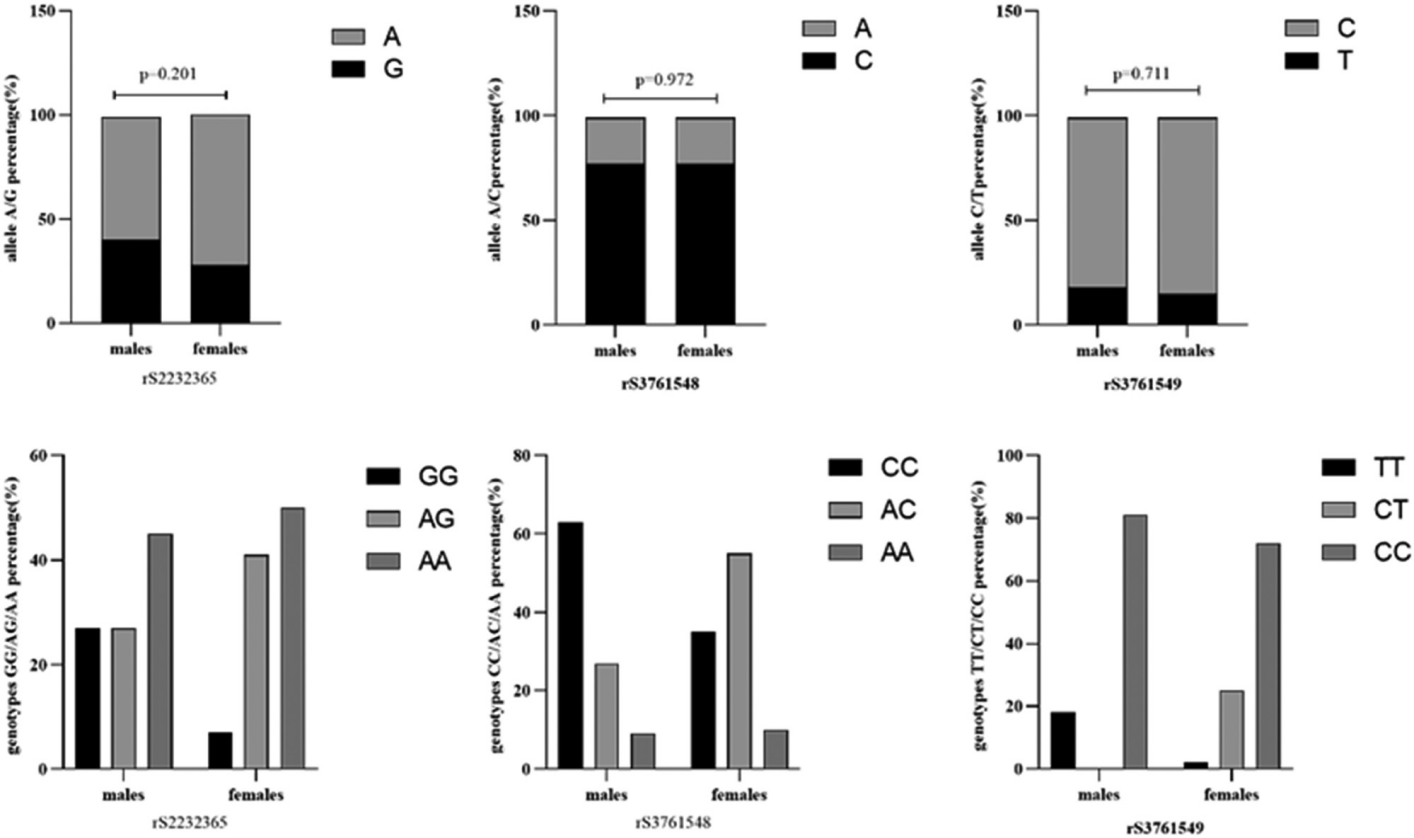
Ratios of alleles and genotypes of rs2232365, rs3761548, and rs3761549 in male and female NMOSD patients. The alleles and genotypes of 11 male and 125 female NMOSD patients, including rs2232365 (A, G), rs3761548 (A, C), and rs3761549 (C, T), as well as rs2232365 (GG, AG, AA), rs3761548 (CC, AC, AA), and rs3761549 (CC, CT, TT), were analyzed. The chi-squared test was applied for the statistical analysis. There was no statistically significant difference in the ratios of the alleles or genotypes of rs2232365, rs3761548, or rs3761549 between male and female NMOSD patients (*p* > 0.05).

Analysis was carried out on 11 male (average age of 45.65 ± 13.88 years) and 125 female (average age of 44.09 ± 5.32 years) NMOSD patients. No obvious differences in age were noted between the two groups (*p* = 0.204). The ratios of the corresponding alleles rs2232365, rs3761548, and rs3761549 were not significantly different between the sexes (*p* = 0.201, *p* = 0.972, and *p* = 0.711, respectively). Figure 5). Specifically, 28.0% of the male NMOSD patients had the G allele of rs2232365, which was lower than the 40.9% ratio of female patients. Analysis of the rs2232365, rs3761548, and rs3761549 genotypes in NMOSD patients of different sexes revealed no statistically significant differences in the rs2232365, rs3761548, and rs3761549 genotypes between male and female NMOSD patients (*p* = 0.074, *p* = 0.092, and *p* = 0.007, respectively) (Figure 5). However, for rs2232365, the GG genotype had a prevalence of 7.1% in females, which was noticeably lower than the 27.3% prevalence in male patients. Similarly, there was a lower percentage of female patients (35.2%) with the CC genotype of rs3761548 than male patients (63.6%). Moreover, for rs3761549, 2.4% of the TT genotype was female, which was markedly lower than the 18.2% ratio in male patients.

## 4 Discussion

In the present study, we elucidated the association between three SNPs (rs2232365, rs3761548, and rs3761549) in *FOXP3* and NMOSD susceptibility in the northern Han Chinese population. We observed that there was no significant association between rs3761548 or rs3761549 and NMOSD risk; however, rs2232365 was significantly associated with decreased NMOSD risk. The MAF of G in rs2232365 was markedly decreased in the NMOSD group compared with the control group, suggesting that rs2232365 allele render protection to NMOSD in this population. In addition, we observed that the GCT haplotype carrying the minor allele G of rs2232365 exerted a protective effect.

*FOXP3* is located on Xp11.23 and is a specific Treg marker. The activities and phenotypes of human Tregs are determined via *FOXP3* expression [32]. During the immune response in humans, a minor CD4 T-cell subset may not lead to an immune response by suppressing additional T-cell functions when they recognize antigens. These cells are called natural Tregs or Tregs [33,34]. CD4CD25 Foxp3 Tregs exert important immunomodulatory effects by secreting immunosuppressive factors such as transforming growth factor-β (TGF-β), IL-10, IL-35, perforin, and granzyme or via cell contact-dependent mechanisms [33,35]. The ability of the immune system to tolerate self- or nonpathogenic non-self-structures can be regulated via immunosuppressive mechanisms. These data suggest that regulating FOXP3 expression can be a critical approach for controlling the immune response. Loss of functional FOXP3 leads to serious autoimmune disorders in patients with immune dysregulation [36,37]. Polymorphisms in *FOXP3* can impair Treg function and result in autoimmune disorders in humans [38]. Abnormal numbers or functional defects of Tregs have been observed in individuals with NMOSD [39]. Therefore, *FOXP3* polymorphisms may be associated with the occurrence of NMOSD via the phenotype and function of Tregs.

NMOSD is a type of CNS autoimmune disorder. However, its pathogenesis has not been fully elucidated, and it has complex genetic and polygenic inheritance patterns. Many genetic models have been examined to explore the relationship between *FOXP3* polymorphisms and NMOSD to better understand the possible genetic patterns of the SNP loci of *FOXP3* during NMOSD. Using codominant, dominant, and recessive genetic models, we observed that patients with the homozygous GG genotype of rs2232365 had a lower NMOSD risk than those with the wild-type AA genotype. Furthermore, the minor allele G of rs2232365 exerts a protective effect on NMOSD, consistent with the findings of Eftekharian et al., in which the FOXP3 rs2232365A/G polymorphism was detected in 410 individuals with MS and 446 healthy controls via PCR-based restriction fragment length polymorphism. They confirmed that *FOXP3* rs2232365 G alleles can predict a markedly decreased risk of MS in the tested population [38]. In addition, Stadtlober et al. included 196 patients with SLE and 157 healthy controls and evaluated the association between rs2232365G/A and rs3761548C/A *FOXP3* variants and SLE susceptibility and the SLE disease activity index; they noted that the G/C haplotype plays a protective role against SLE, whereas allele A in the above two variants may favor disease activity and autoimmunity. *FOXP3* variants may affect Treg activity by inhibiting TGF-β1 generation and increasing disease activity and autoantibody levels in patients with SLE [40]. This finding is similar to that of our study in that the G haplotype (GCT) carrying the minor allele of rs2232365 may exert a protective effect. In the future, we should further investigate the specific mechanism underlying the minor allele GCT of rs2232365 in NMOSD. Interestingly, in another study that included 50 patients with Crohn’s disease (CD) and 154 healthy controls, the rs2232365 haplotype (dominant model) exerted a protective effect on 60% of patients with CD susceptibility and decreased the endoscopic severity index by 60% [41]. In addition, the rs2232365A/G FOXP3 variant is associated with prognostic outcomes and susceptibility to different autoimmune disorders, such as RA [42,43]. These findings support the findings of our study. These results revealed that FOXP3 polymorphisms are associated with the susceptibility of NMOSD in the Northern Chinese Han population.

FOXP3 exerts an important regulatory effect on FOXP3+ Tregs. Diverse single-nucleotide variants have been detected in the FOXP3 promoter region; these variants affect *FOXP3* expression while impairing Treg differentiation and function, further affecting the occurrence and development of autoimmune diseases [44,45].

Subsequently, we stratified AQP4-IgG status and onset symptoms according to the rs2232365, rs3761548, and rs3761549 genotypes. However, these three SNPs were not associated with NMOSD onset symptoms or AQP4–IgG status. These results can shed novel insights into the relationship between FOXP3 polymorphisms and NMOSD.

However, the classification of patients with NMOSD into moderate-to-severe (EDSS ≥3 points) and mild (EDSS <3 points) groups for the three genotype frequencies of rs2232365 resulted in significant differences between the two groups (*p* = 0.001). In the moderate-to-severe group, the frequency of allele AA was 64.3%, which was significantly greater than that in the mild group (36.4%). In contrast, in the mild group, the frequency of the genotype GG was 15.2%, which was significantly greater than that in the moderate-to-severe group (2.9%). Therefore, the GG genotype may decrease NMOSD severity, whereas the AA genotype may be associated with NMOSD severity. This finding is similar to the findings of Stadtlober et al., who indicated that the allele A of rs2232365 may favor disease activity and autoimmunity [40]. In future studies, the sample size should increase, and the genotype should be further stratified to explore and identify the associations between disease severity and pathological mechanisms.

Next, we assessed the possible relationships between the three *FOXP3* SNPs and mRNA expression. In our study, *FOXP3* mRNA expression did not significantly differ among patients with NMOSD of diverse genotypes. However, *FOXP3* mRNA expression was lower in patients with NMOSD and carrying the AA genotype of FOXP3 rs2232365 than in those carrying the AG or GG genotype; on the other hand, *FOXP3* mRNA expression was greater in patients with the GG genotype than in those with the AG or AA genotype. The rs2232365 genotype was correlated with *FOXP3* mRNA expression in the following order: GG > AG > AA. Furthermore, the results regarding the sex distribution of the FOXP3 polymorphism showed that for rs2232365 in females, the G allele and GG genotype were significantly lower than those in males, the CC genotype at rs3761548 was lower than that in males, and the TT genotype at rs3761549 was lower than that in males. These observations suggest that alterations in the alleles and genotypes of rs2232365, rs3761548, and rs3761549 may have greater potential to influence the expression level of female FOXP3 mRNA, which in turn may have an impact on the onset of NMOSD in female patients. Although the above experimental results showed no significant difference, other related studies have shown that gene polymorphisms might regulate mRNA expression through various mechanisms [56], and the degree of expression of SNPs correlates with the level of the inactive X chromosome [57]. Additionally, some genes with SNP–sex interactions exhibit sex-biased gene expression [58]. In future studies, the sample size should be expanded to further elucidate the relationships among FOXP3 polymorphisms, sex, and mRNA expression, as well as research into SNP functionality. Finally, we determined *FOXP3* mRNA expression in 63 patients with NMOSD and 35 healthy controls and observed that *FOXP3* mRNA expression was increased in the NMOSD group compared with the control group (*p* = 0.001). Among the 63 NMOSD patients, 57 underwent immunotherapy-related treatment, which could lead to an increase in FOXP3 mRNA and thus facilitate the repair of immune regulation. This finding is in line with the findings of Pitarokoili et al. [54,55], who suggested that FOXP3 mRNA expression can be upregulated after treatment with immunotherapy-related drugs. However, these findings contradict those of Brill et al. [29]. Therefore, the sample size needs to be increased to investigate the expression of FOXP3 mRNA further. Considering that there is a drug effect, patients in the acute phase were divided into three groups: the ANT, AOGC, and remission groups. *FOXP3* mRNA expression was lower in the ANT group than in the control group (*p* = 0.004). Furthermore, compared with that in the AOGC and remission groups, *FOXP3* mRNA expression was significantly lower in patients with NMOSD (*p* = 0.009). In addition, the ANT group exhibited significant differences compared with the remission group (*p* = 0.007). *FOXP3* mRNA expression was increased in the AOGC group compared with the ANT group but decreased compared with that in the remission group, but the difference was not statistically significant. This may be due to the small sample size of our study. Subsequently, *FOXP3* mRNA expression was markedly decreased in the ANT group. Therefore, *FOXP3* mRNA expression has some significance for predicting disease activity and evaluating therapeutic effects. In the present study, we detected *FOXP*3 expression in PBMCs collected from patients with NMOSD and compared it between the ANT and control groups for the first time. Because the sample size of our study was small, future studies should be conducted with a larger sample size; in addition, additional mechanistic studies are warranted, and the effects of drug treatments should be considered.

## 5 Conclusion

We observed that *FOXP3* polymorphisms and haplotypes are associated with NMOSD susceptibility in the Han Chinese population. The minor G allele of *FOXP3* rs2232365 and the GCT haplotype (the rs2232365, rs3761548, and rs3761549 alleles) are associated with decreased NMOSD susceptibility and protection against NMOSD in this population. Furthermore, the GG genotype may decrease the severity of NMOSD, whereas the AA genotype may be associated with moderate NMOSD severity. *FOXP3* mRNA expression was lower in the ANT group than in the control group. These results suggest that *FOXP3* is a candidate mechanism-specific druggable target for NMOSD and has potential value for observing the dynamics of this disease. Nevertheless, our sample size is insufficient to achieve the ideal statistical power needed to detect correlations of small effect sizes; therefore, our findings should be further validated in larger cohorts. Moreover, we should determine the effect of rs2232365 on the pathogenesis of NMOSD, which is our future research direction.

## Supplementary Material

supplementary material
