# Supplementary material for: Association between FOXP3 polymorphisms and expression and neuromyelitis optica spectrum disorder risk in the Northern Chinese Han population
Source: Transl Neurosci. 2024 Apr 5;15(1):20220337. doi: 10.1515/tnsci-2022-0337 (PMC10998649; doi:10.1515/tnsci-2022-0337)
Supplement: supplementary material [file tnsci-2022-0337-sm.pdf]

Supplementary material

Primer information.

Gene	Forward (5′ → 3′)	Reverse (5′ → 3′)
Foxp3	CAAGGAAAGGAG GATGGACGAACAG	GGCAGGCAAGACA GTGGAAACC
GAPDH	GGAAGCTTGTCA TCAAT	TGATGACCCTTTTGGCT

S1 SNP Genotyping experiment process

1. PCR Amplification: Divide the prepared PCR total volume into a 96-hole PCR board, centrifuge it, add 1 μl DNA to each hole, centrifuge it again, and put it on a PCR device. The PCR reaction system is as follows:
   DNA 1 μl
   10*buffer 1.5 μl
   MgCL2 (25 mmol) 1.5 μl
   DNTP(10 mmol) 0.3 μl
   Primer (10 μmol) 0.15 μl/branch
   Taq Enzyme (5 μ/ μl) 0.3 μl
   H2O Total volume to 15 μl
   Amplification conditions:
   94℃ 3 min
   94℃ 15 s
   55℃ 15 s For 30 cycles
   72℃ 30 s
   72℃ 3 min

Table S2: Demographics and clinical characteristics of participants for *FOXP3* SNP

	NMOSD	HCs	*P*-values
	n = 136	*n* = 224	
Sex, no. (%) of females	125 (91.9)	207 (92.4)	0.864
Age, *y* (mean ± SD)	45.53 ±14.15	43.77 ±11.60	0.224
Age at onset, *y* (mean ± SD)	40.58±12.96	NA	NA
AQP4-IgG$^+$, no. (%) of patients	107 (78.68)	NA	NA
Onset symptoms, no. (%) of patients			
Optic neuritis	52 (38.24)	NA	NA
Acute myelitis	57 (41.91)	NA	NA
Brain attacks	16 (11.76)	NA	NA
Mix attacks	11 (8.09)	NA	NA
Family history	NA	NA	NA

Abbreviations: NMOSD, neuromyelitis optica spectrum disorders; HC, healthy controls; SD stands for standard deviation; NA stands for data not available. Brain involvement includes the impairment of the brainstem, cerebral hemispheres, or cerebellum.

Table S1: Primers used in the genotyping of single-nucleotide polymorphism in the *FOXP3* gene by SNaPshot

Gene	Primer sequence		Position
	Upstream primer (5′)	Downstream primer (3′)	
rs2232365	TCAAGGTGAGGACAATAGAAGAGC	GGAGGCGAGTCCAGGAGTGT	Chr X:49259429 (GRCh38.p14)
rs3761548	TTGAAGACCAGAGATCTCAGGG	TGTGATCGTGGATCGTCCAAC	Chr X:49261784(GRCh38.p14)
rs3761549	TGGCACTCTCAGAGCTTCAAAC	AGGAAGAGAAGAGGGCAGATAC	Chr X:49260888(GRCh38.p14)

2. PCR product purification

After PCR amplification, take 3 µl PCR products and purify them with ExoI and FastAP. This is mainly to remove the remaining primers in the reaction product with ExoI and the remaining DNTPs in the reaction with FastAP.

PCR product 3 µl
ExoI (20 µ/ µl) 0.2 µl
FastAP(1 µ/ µl) 0.8 µl
ExoI buffer 0.7 µl
H2O Total to 7 µl

37°C 15min, 80°C 15min, perform the extension reaction after purification is complete and have extension primer mixed already.

3. Extension reaction

System:
PCR Product 2 µl
Snapshot Mix Reagent 1 µl
Extension Primer(10 µmol) 0.2 µl/branch
Water to 6 µl
Conditions:
96°C 1min
96°C 10 s
52°C 5 s For 30 cycles
60°C 30 s

Take 1 µl of extension product, add 9 µl HID denaturation buffer, denature at 95°C for 3min, immediately place in an ice bath, and put it on a sequencer.

Representative Peak Chart：A(green), G(blue), C(black), T(red)

Sequence number	Rs_ num	PCR product length and GC content	SNPs	Extension direction	Extended products	Extension primer length
J463-S4	RS3761548	234; 56.8%	[A/C]	F	AC	35
J463-S5	RS2232365	246; 56.9%	[A/G]	F	AG	45
J463-S6	RS3761549	216; 58.3%	[C/T]	F	CT	42

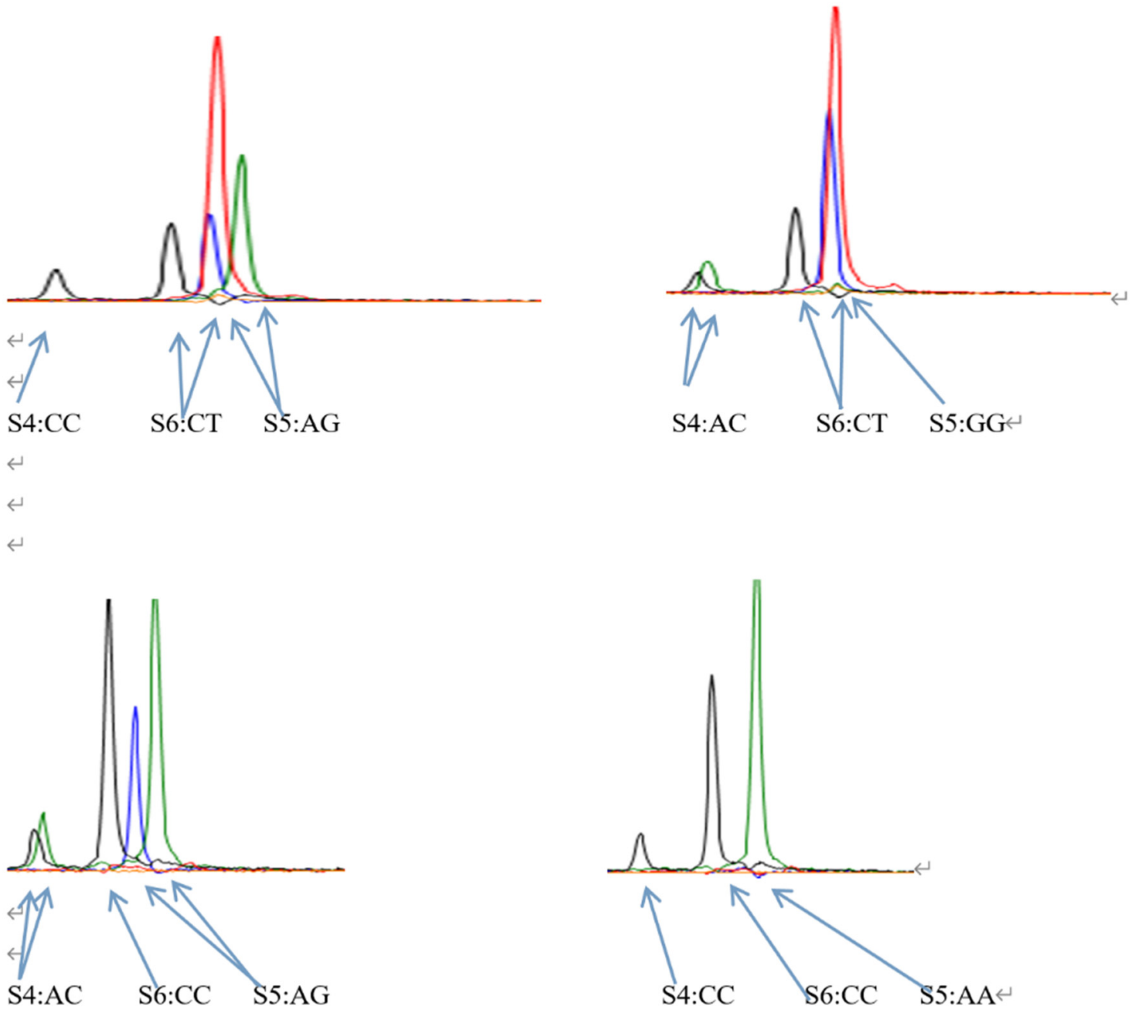

S5:RS2232365

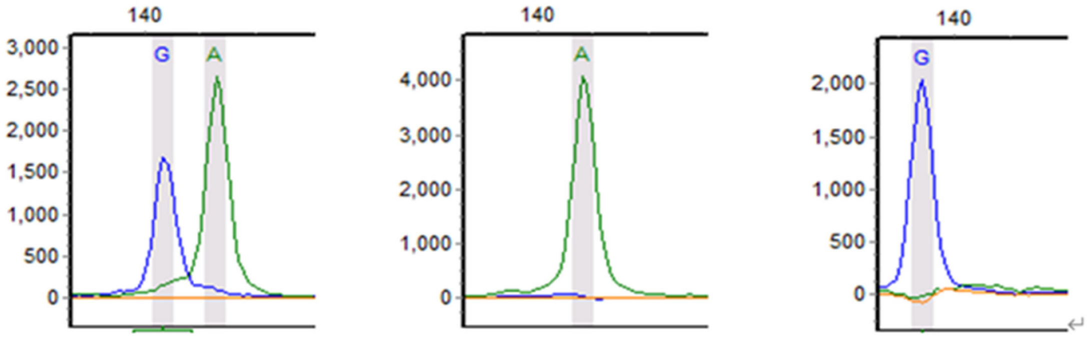

S4:RS3761548

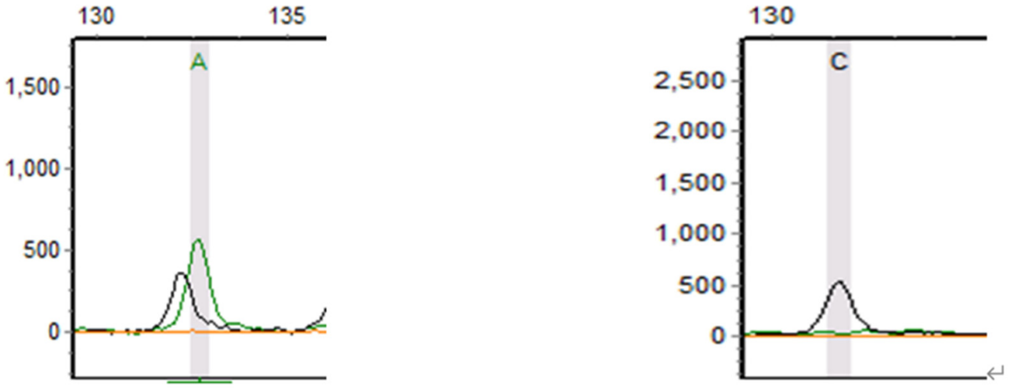

S6:RS3761549

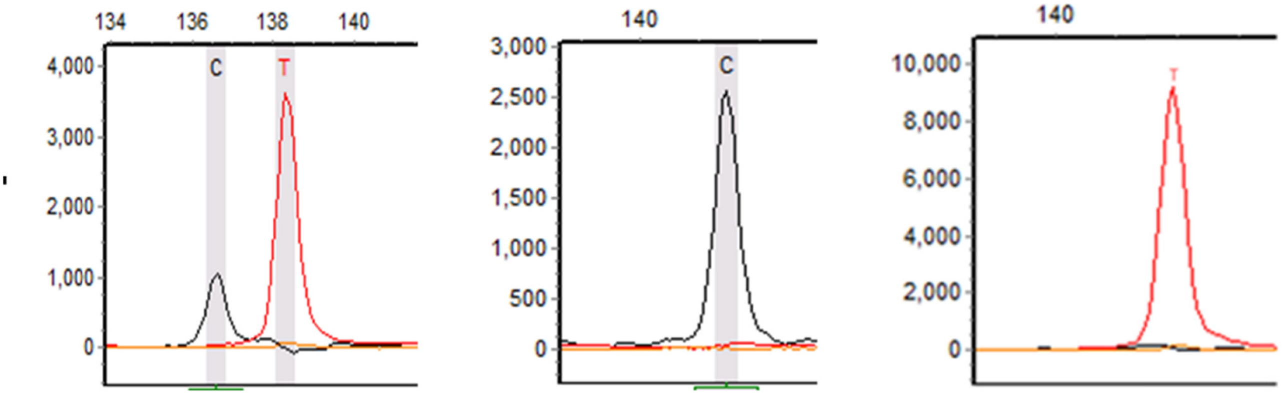

Representative images showing different peak heights of the alleles of rs2232365, rs3761548, and rs3761549, with a single peak representing a homozygote while a double peak represented a heterozygote.
